# A Lack of Focus, Not Task Avoidance, Makes the Difference: Work Routines in Procrastinators and Non-Procrastinators

**DOI:** 10.3390/bs13040333

**Published:** 2023-04-15

**Authors:** Francesco Di Nocera, Rosa De Piano, Marika Rullo, Giorgia Tempestini

**Affiliations:** 1 Department of Planning, Design, and Technology of Architecture, Sapienza University of Rome, 00196 Rome, Italy; 2 Department of Psychology, Sapienza University of Rome, 00185 Rome, Italy; 3 Department of Social, Political, and Cognitive Sciences, University of Siena, 53100 Siena, Italy

**Keywords:** behavior, procrastination, stress, coping, knowledge work

## Abstract

Procrastination may be seen as the outcome of a learning history of delaying the onset of task execution and its completion, both in terms of time and effort. In this study, we examined the performance of 55 university students who carried out two writing tasks consisting of summarizing two academic papers, each within a different time slot (i.e., five vs. three days to complete). The two assignments were part of the class activity and were perceived by participants as homogeneous in terms of text appreciation and difficulty, therefore making the two conditions comparable. The Pure Procrastination Scale was used to categorize subjects as high and low procrastinators, and to compare their performances. Results show that students who report more procrastination behaviors tend to increase their productivity as the deadline approaches, while low procrastinators are more productive throughout the time at their disposal, with peak activity during the intermediate day. Such a strategy was consistent across two deadlines (five vs. three days), and the difference between the two subgroups can be ascribed to the task-oriented coping style, which seems to be lacking in high-procrastinators.

## 1. Introduction

Procrastination is a complex phenomenon involving cognitive, emotional and behavioral components [1], see also [2], that is generally described as postponing an intended course of action. This phenomenon has been widely analyzed in the past, though it continues to be an object of study nowadays, where increased production requires stricter schedules in order to meet demand, therefore discouraging delay [3]. The common view of procrastination is that people engage in such behavior intentionally, even if they are aware of its potential negative consequences [4,5,6,7,8,9,10]. The negative consequences concern many dimensions, such as performance [11], subjective well-being [12] and even physical health [13]. Moreover, procrastination is a widespread phenomenon, with evidence revealing that it affects 75% of students and approximately 15–20% of adults worldwide [4,14,15]. Specifically, academic procrastination is mainly associated with high stress levels, guilt, low self-esteem, anxiety, and depression (see [16,17]), often inducing students to experience academic burnout [18,19].

Recent studies have dealt with this phenomenon in students during the COVID-19 pandemic, identifying increased procrastination behaviors because of isolation. Students experienced increased anxiety, fear, stress and worry due to the need to reorganize time and due to the increased time spent using electronic devices and consuming social media consumption [15,20].

Given its diffusion and its negative impact on life, many researchers have investigated this phenomenon closely. A seminal work by Solomon and Rothblum [21] suggested that people likely delay completing (aversive) tasks due to their fear of failure and the anxiety associated with such a belief. Other studies have suggested that procrastination is a time management issue that comprises difficulties related to planning and managing an intended course of action (see [22] for a discussion). Ferrari [23,24,25] highlighted three kinds of procrastination: decisional (a cognitive component referring to the individual’s inability to promptly come to a decision), avoidant (a coping strategy people use to avoid aversive tasks), and arousal (a task performance approach that involves postponing a task until the last minute available).

Considering the ubiquity of procrastination across time and situations, many authors have focused their attention on the individual differences most frequently associated with procrastination when it is viewed as a ubiquitous disposition [4,26].

Procrastination is often correlated with low levels of conscientiousness [27], a lack of time management skills, low self-efficacy, and low self-esteem [28,29,30,31,32,33]. Although the literature has placed the focus primarily on these traits, procrastination seems to be a (presumably learned) behavioral strategy that is used to avoid the unpleasant feelings associated with the completion of aversive tasks, such as difficult or boring tasks [26,34,35], which are often thought of as not interesting or unrelated to personal development [36]. These ideas align with previous research showing that individuals with higher levels of work engagement, defined as a positive and fulfilling work-related state of mind characterized by vigor, dedication, and absorption [37], may be less likely to procrastinate. Indeed, recent research has shown that work engagement is positively associated with task performance [38,39]. In addition, other factors, such as autonomy and mastery, can influence work engagement and predict better performance outcomes [38,39,40]. Thus, more engaged individuals may be more likely to experience intrinsic motivation and a sense of purpose, making it easier to overcome the desire for immediate gratification and stay focused on long-term goals. This idea is consistent with the evidence of links between procrastination and impulsivity, where individuals tend to prefer smaller and sooner rewards over larger and later rewards [28,29].

Somehow, individuals may use procrastination deliberately to tackle everyday tasks, such as when they perceive the pressure of a close deadline as a motivational boost that increases their productivity (e.g., [6,41,42,43]). Early studies from Schouwenburg and colleagues [28,44,45] revealed that procrastination strictly depends on temporal discounting [46,47,48]. This principle implies that when the potential rewards associated with an activity are too distant in time from the activity itself, they are not attractive to sustain the activity. Speaking of the academic context, passing an exam (potential reward) implies that you have to study (activity). According to the discounting principle, if the exam date is scheduled for a long time in the future (e.g., two months), students will postpone studying until the potential reward is closer.

This attitude easily permits the association between the activity and its potential reward (consequence), likely sustaining the coherent behavior needed to achieve the reward. In this regard, previous studies have confirmed that procrastination is a dynamic behavior that follows a curvilinear trajectory over time (e.g., [49]), with a sharp decrease in procrastination and increased task-related behaviors as the deadlines associated with the tasks approach. This pattern of behavior also seems to be quite similar both among high and low procrastinators [50]. This evidence has also been recently supported in a study by Zhang and Feng [51], who proposed a temporal decision model to explain procrastination. The authors argue that people procrastinate until the avoidance of an activity exceeds the benefits it can produce. In particular, people perceive an activity as less aversive when the deadline is far away in time and not scheduled in the immediate present. At the same time, they expect the benefits to be higher in the case of long-term rather than short-term tasks. 

Overall, procrastination seems to result from a complex balance among self-control, emotional regulation, and the characteristics of the potentially postponed tasks. These factors together may contribute to the persistence of a procrastination strategy over time. 

Strictly related to the scope of the present work, we consider procrastination as the outcome of a learning history that sees the delay of the onset of task execution and its completion in terms of time and effort. In particular, we look at procrastination as a strategy that consists of postponing the work to do until the last minute available (e.g., the day before or the same day of the deadline). Therefore, we investigated the ongoing productivity of a sample of students to gather information about when students concentrated the bulk of their activity regarding an assigned task. Students’ productivity was assessed both with objective (i.e., the amount of time and keystrokes) and subjective (i.e., self-report questionnaires) measurements. Many authors question the utility of relying exclusively on trait-based self-report measures of procrastination, which sometimes have been found to be unrelated to actual procrastination behavior [4]. In this respect, our research strategy overrides the drawbacks of exclusively implementing self-report measures of task completion [52]. Nevertheless, since we consider procrastination as a behavioral strategy, we also employed the Pure Procrastination Scale [4]. This is an inventory of procrastination behaviors that people rate in frequency, and that was found to be a predictive measure of actual procrastination in a recent work by Zuber et al. [53]. Moreover, given that procrastination has been associated with a lack of emotion regulation, we decided to use the Coping Inventory for Stressful Situations (CISS) to assess the impact of the coping strategies that people generally adopt to deal with stressful (i.e., aversive) events. CISS is a self-assessment questionnaire based on three kinds of coping strategies: (i) task-oriented; (ii) emotion-oriented; and (iii) avoidance-oriented strategies (more details in the Measures section). The likelihood of adopting one coping strategy over another might be the consequence of the individual’s experience. In their learning history, individuals might have found, for example, the task-oriented approach to be the most appropriate behavior to overcome aversive tasks and reach goals.

Other procrastination studies have attempted to objectively measure task performance, but used a narrower focus on simple one-shot tasks that failed to capture those task characteristics mainly related to procrastination. More specifically, simple one-shot tasks reveal nothing specific about the timeline of an individuals’ effort when dealing with a task. They cannot provide information about the ongoing attempts to work on the assigned task. Therefore, we aimed to measure ongoing productivity by analyzing the number of keystrokes and the time spent on a writing task, namely summarizing academic articles. Thus, the data flow depicts a continuous process in which the individual may work for different amounts of time during the assigned period.

Such a research strategy allows a more realistic analysis of what occurs in many cognitively relevant tasks (e.g., creative and intensive cognitive activities) in academic and working environments. Procrastination is indeed an issue in the work domain, particularly for activities involving knowledge production. Academia is the occupation that is most affected by the issue of procrastination because the goal of the work activity is usually reached over a relatively long time (from days to months). In contrast, other work activities may have immediate consequences. For example, a manufacturer worker cannot procrastinate because any delay would be immediately recognized as a violation. 

### Purpose of the Current Study

The aim of this experimental study is threefold. First, we examine how students deal with real academic tasks associated with deadlines by observing when, how much, and how long they work. In this way, we can track the development of the procrastination that is often observed among people who allow most of the work to be completed near the given deadline. We relied on a self-reported procrastination scale (PPS, [4]) to determine the behavioral patterns of people with differential levels of procrastination. The PPS was administered later to avoid priming effects.

Our first hypothesis was that procrastinators and non-procrastinators would show different productivity patterns along the timeline, leading to two different productivity curves.

The second aim was to verify whether shortening the deadline affects procrastination and whether this would affect high and low procrastinators differently. To do that, we experimentally introduced two schedules in which to deliver the assignment of the two writing tasks to all participants. The first schedule was based on five days, while the second was on three days. Introducing this change in the schedule allowed us to explore whether the procrastination curve would change. We expected it to be steady and “high” when the deadline was very close to when the assignment was given, especially among high procrastinators. 

Accordingly, our second hypothesis was that shortening the deadline would affect procrastinators’ and non-procrastinators’ productivity patterns differently.

Finally, we aimed to investigate the role of the coping strategy in differentiating between high and low (self-defined) procrastinators, assuming that high procrastinators are less task-oriented and more avoidance-oriented than low procrastinators. The CISS was administered afterward to avoid priming effects.

Hence, our third hypothesis was that the coping strategies adopted by procrastinators and non-procrastinators would differ, therefore accounting for the differences in the participants’ behavioral strategies when dealing with the stressful experience of working to meet a deadline.

As an accessory aim, we also wanted to provide further evidence on the predictive ability of a self-reported procrastination scale such as the Pure Procrastination Scale [4] by comparing this self-report measure with the actual behavior.

## 2. Materials and Methods

### 2.1. Participants

The sample comprised 55 university students (mean age = 24.61; st. dev. = 4.57; 44 females) regularly enrolled in a course on a second-year Master’s program at the Sapienza University of Rome with mandatory attendance. The gender imbalance reflects the composition of undergraduate courses in Psychology at our university. All students authorized the use of the data collected during the course activity for research purposes. 

### 2.2. Research Design

**Figure 1:**
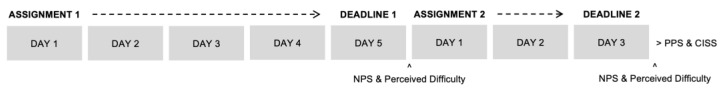
Overview of the study procedure: Participants were asked to summarize two academic articles in two different sessions. The deadline for the first session was set to 5 days, whereas that for the second session was set to 3 days. The NPS and a set of items for assessing the perceived difficulty of the task were administered after each session. The PPS and the CISS questionnaires were administered at the end of the study.

The study was based on two mixed designs in which high and low procrastinators (defined according to the subjects’ score to a procrastination scale) performed two equivalent writing tasks: one with 5-day deadline and another with a 3-day deadline (see overview in Figure 1). Therefore, the procrastination profile was the fixed factor and the day of writing was the repeated factor.

### 2.3. Experimental Procedure

Participants were asked to summarize two academic articles in at least three standard pages of 1800 characters each (spaces included) by the deadline set for each task. As reported above, the first deadline was set to 5 days, whereas the second was set to 3 days. Tasks were performed one week apart and were embedded into the course activities. 

The text to be summarized (a unique article for each participant in each session) was emailed in PDF format. Each participant was asked to write the synthesis using a writing platform coded specifically for this study. The writing platform recorded all the typing produced by each participant during the entire study duration. At the beginning of the study, each participant received a link to access their user area on the platform. The user area consisted of a blank page where subjects could write. Pasting from other documents was not allowed by the application. An indicator of the amount of work done (standard pages produced) was always available at the top of the interface.

The texts selected for each task dealt with the course topic and were homogeneous in terms of length and language complexity. They were all English-written academic articles from scientific journals and edited books.

After completing each task, participants were asked to fill out a task evaluation questionnaire, answering questions about the perceived difficulty and enjoyability of the task. Finally, two days after the completion of the last writing task, participants were asked to respond to the Pure Procrastination Scale (PPS) (PPS; [4,54]) to assess self-reported procrastination, and the Coping Inventory for Stressful Situations (CISS) [55] to evaluate their coping strategy.

### 2.4. Measures

Both self-report measures and objective measures were used to assess procrastination. As previously mentioned, with regard to the subjective measures, three questionnaires were administered to the participants: the PPS, the CISS, and a task evaluation survey.

*Self-report Procrastination*: To assess self-reported procrastination, we relied on the Italian version of the PPS Pure Procrastination Scale [56,57], which consists of 12 items on a 5-point Likert scale used by participants to indicate how accurately some statement described their habits (1 = “very seldom or not true for me”; 5 = “very often true for me”). 

*Coping strategy*: The questionnaire used to assess the coping strategies was the Italian version of the Coping Inventory for Stressful Situations (CISS: [55,58], a multidimensional tool to measure the individual coping style, consisting of 48 items that refer to three different coping dimensions: (i) Task-oriented coping, in which the focus is on the task and the effort to solve stressful situations; (ii) Emotion-oriented coping, in which the emphasis is on the self-oriented emotional reactions of the individual; and (iii) Avoidance-oriented coping, which refers to activities and cognitive strategies put in place to avoid upsetting events. Subjects had to evaluate how precisely each statement represented them or their behaviors when facing stressful or difficult situations. The assessment of the items was based on a 5-point Likert scale, from 1 = “very seldom or not true for me” to 5 = “very often true for me”. 

Additional measurements and indexes were calculated considering the number of characters (spaces included) recorded by the writing platform and the items included in the evaluative questionnaire the participants had to fill out at the end of each writing task. The following paragraphs describe the measures mentioned above in detail.

*Appreciation of the topic:* the Net Promoter Score (NPS: [59,60] is a tool adopted to assess customer satisfaction. It comprises one single item: “How likely would you recommend this product to a friend or colleague?”. Respondents have to rate the likelihood that they would recommend a product/a service or a company to another person; the rating scale goes from 0 to 10. People with scores between 9 and 10 are classified as “promoters”; those with a score of 7 to 8 are defined as “passives”; while responses between 0 and 6 fall into the category of “detractors”. In this experimental study, we reformulated the single item of the NPS as follows: “How likely would you recommend this paper to a friend or colleague?“. In this study, the raw score (0 to 10) was used as a dependent variable for assessing the potential effect of the appreciation of reading and summarizing the article.

*Perceived difficulty:* The perceived difficulty was assessed with four items asking “How difficult did you find the article in relation to language (English)/content/length” from 1 = “not difficult at all” to 5 = “completely”, and the appropriateness of the deadlines “How adequate did you find the deadline provided for the completion of the task”? from 1 = “totally inappropriate” to 5 = “totally appropriate”.

*Productivity indices for the study/writing assignments:* For each assignment, we calculated the time they spent on the writing task (both daily and globally) and the number of keystrokes.

- Productivity (keystrokes): We used the proportion of keystrokes produced on each working day relative to the total keystrokes made for each assignment (5 or 3 days). Keystrokes included deletion, as it is always a production.
- Productivity (time on task): We used the proportion of time devoted to the writing task on each working day relative to the total time dedicated to each assignment (5 or 3 days).

## 3. Results

The sample size in this study could not be controlled by experimenters as it was conditioned by the number of students who were enrolled in the course. However, a post-hoc power analysis showed that the sample size for the design that required the largest number of subjects reached a power value of 0.9 for a medium effect size (Cohen’s *f^2^* = 0.3).

Prior to analyzing the participants’ productivity, we ran a set of control analyses to check whether the task assignments were homogeneous between the two schedules.

The NPS score was used as a dependent variable in a repeated measure ANOVA design, employing the schedule (five days vs. three days) as a repeated factor. Results showed that the schedule had no significant effect on text appreciation (F_1,54_ = 0.195, *p* > 0.05). 

The same analysis, using the four dimensions related to the perceived difficulty (i.e., content, length, language, adequacy of time) of the reading materials, revealed that the content (F_1,54_ = 0.053, *p* > 0.05), the length (F_1,54_ = 0.225, *p* > 0.05) and the language (F_1,54_ = 0.857, *p* > 0.05) were not statistically different between the schedules. As expected, we found a significant effect on the perception of the adequacy of the time provided for the assignment (F_1,54_ = 34.686, *p* < 0.001, η_p_^2^ *=* 0.391): the time provided was perceived as less adequate to complete the task when the schedule for the assignment was settled after 3 days with respect to the 5-day assignment.

### 3.1. Productivity—Keystrokes

The proportion of keystrokes (angular transformation) produced by the participants was used as a dependent variable in a mixed ANOVA design employing the procrastination profile (Low vs. High) as a fixed factor and the day of writing (Day 1 vs. Day 2 vs. Day 3 vs. Day 4 vs. Day5 ) as a repeated factor. The results showed no significant effect of the profile (F_1,53_ = 3.102, *p* > 0.05), the significant main effect of the day (F_4,212_ = 9.485, *p* < 0.001, η_p_^2^ = 0.152) and the significant interaction between the profile and day (F_4,212_ = 2.879, *p* < 0.05, η_p_^2^ = 0.051). Post hoc analyses (Duncan test) of the interaction showed that the two groups were significantly different on Day 3 and Day 5, respectively. Specifically, low procrastinators showed a peak of performance on Day 3, whereas the high procrastinators showed a peak of performance on Day 5. 

**Figure 2:**
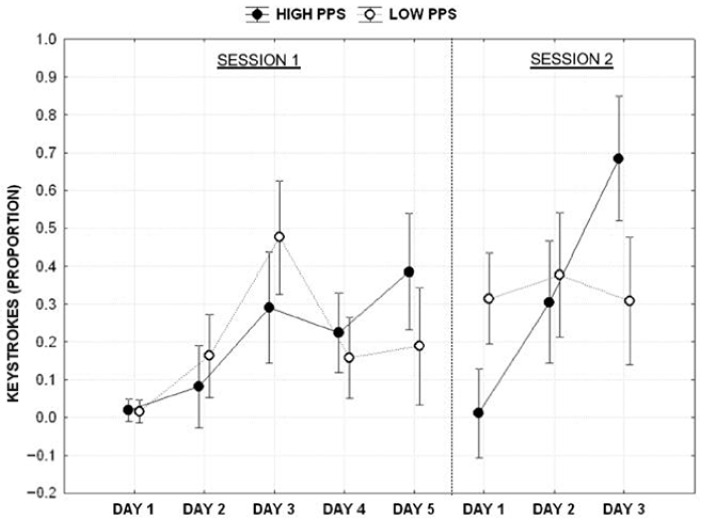
Proportion of keystrokes (text production) separately per session, day, and category of subjects. Vertical bars denote 0.95 confidence intervals.

The same analysis was performed using the day of writing for the 3-day deadline condition (Day 1 vs. Day 2 vs. Day 3) as a repeated factor. The results showed no significant effect of the profile (F_1,53_ = 0.963, *p* > 0.05), the significant main effect of the day (F_2,106_ = 7.274, *p* < 0.001, η_p_^2^ = 0.121) and the significant interaction between the profile and day (F_2,106_ = 7.811, *p* < 0.001, η_p_^2^ = 0.128). A post hoc analysis (Duncan test) of the interaction revealed that low procrastinators consistently performed during the three days with no statistically significant difference between days. In contrast, the high procrastinator group showed a significant linear increment across work days from the first to the third (Figure 2).

### 3.2. Productivity—Time Spent on the Task

Concerning the amount of time spent on the task, which was used as a dependent variable, a mixed ANOVA that employed the procrastination profile (Low vs. High) as a fixed factor and the day of Writing (Day 1 vs. Day 2 vs. Day 3 vs. Day 4 vs. Day 5) as a repeated factor revealed no significant effect of the profile (F_1,53_ = 2.047, *p* > 0.05), the significant main effect of the day of writing (F_4,212_ = 10.058, *p* < 0.001, η_p_^2^ = 0.159) and the significant interaction between the profile and day (F_4,212_ = 4.097, *p* < 0.05, η_p_^2^ = 0.072). Post hoc analyses (Duncan test) of the interaction showed that the two groups were significantly different on Day 3 and Day 5, respectively. Specifically, low procrastinators showed a peak of performance on Day 3, whereas high procrastinators showed a peak of performance on Day 5. 

**Figure 3:**
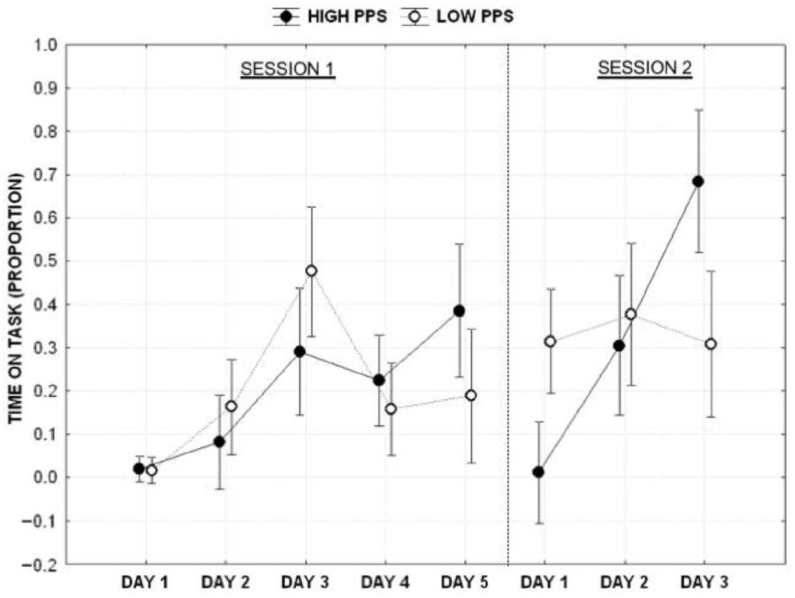
Proportion of time dedicated to the writing task separately per session, day, and category of subjects. Vertical bars denote 0.95 confidence intervals.

The same analysis performed on the 3-day deadline confirmed no significant effect of the profile (F_1,53_ = 0.964, *p* > 0.05), the significant effect of the day (F_2,106_ = 8.253, *p* < 0.001, η_p_^2^ = 0.135) and the significant effect of the interaction between the profile and day (F_2,106_ = 9.252, *p* < 0.001, η_p_^2^ = 0.149). A post hoc analysis (Duncan test) of the interaction revealed that low procrastinators showed consistent performance during the three days with no statistically significant difference between days. In contrast, the high procrastinator group showed a significant linear increment across work days from the first to the third (Figure 3).

### 3.3. Coping Strategy

**Figure 4:**
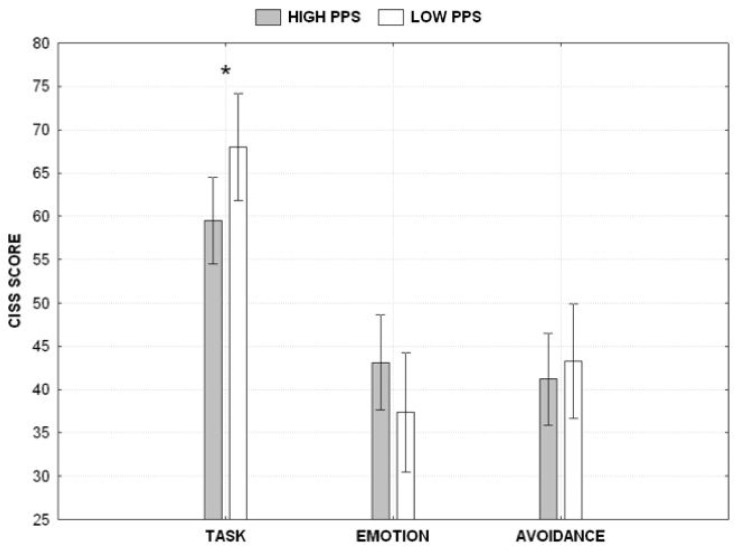
CISS score separately per scale (task-oriented, emotion-oriented, avoidance-oriented) and category of subjects. The asterisk denotes a statistically significant difference. Vertical bars denote 0.95 confidence intervals.

We performed another mixed ANOVA by employing the profile as a fixed factor and the sub-dimensions of the CISS scale as repeated factors (task-oriented vs. emotion-oriented vs. avoidance-oriented). Results revealed no significant effect of the profile (F_1,53_ = 0.898, *p* > 0.05), the significant main effect of the sub-dimensions (F_2,106_ = 51.596, *p* < 0.001, η_p_^2^ = 0.493) and the significant interaction between the profile and sub-dimensions (F_2,106_ = 6.300, *p* < 0.01, η_p_^2^ = 0.106). A post hoc analysis (Duncan test) revealed that low procrastinators reported significantly higher scores on the task-oriented dimension than high procrastinators, while all the other differences were not statistically significant (Figure 4).

## 4. Discussion and Conclusions

Contemporary life is complex. We are overwhelmed by a variety of sources of information to pay attention to, roles to play, and tasks to accomplish. Isolating the effects of the myriad of factors that affect our ability to meet deadlines is inconceivable. Despite this variability, some people consistently seem to struggle more than others to be complete tasks. The present study examined the time course of procrastination using an academic task and manipulating the time allowed to complete the assignment. We aimed to investigate how people procrastinate and to give an insight into how this behavior evolves. To achieve this goal, we asked students of a Master’s program course to perform two writing tasks, summarizing two academic papers, each with a different schedule (i.e., five vs. three days to complete). Our participants were final-year students (ready to go to work) and thus fully representative of the type of population affected by the problem of procrastination in academia. In addition, it is worth noting that students attended the same class, which made it possible to have both a homogeneous sample and a homogeneous workload (since, at least from the point of view of academic tasks, they were all subjected to the same demands). The two assignments were part of the class activity and were perceived by participants as homogeneous in terms of text appreciation and difficulty, making the two conditions comparable. The time provided for accomplishing the task in 3 days was perceived as less adequate than the 5-day deadline. Therefore, participants could acknowledge the time pressure differences between the two conditions. The Pure Procrastination Scale questionnaire [4] was used to categorize subjects as high and low procrastinators and to compare their performances.

The results show that people who report more procrastination behaviors tend to increase their productivity as the deadline approaches. At the same time, low procrastinators are more productive throughout the time at their disposal, with peak activity during the intermediate day. This result supports our first hypothesis: procrastinators and non-procrastinators show different productivity patterns along the timeline, leading to two different productivity curves. We also found that such a strategy was consistent across two types of deadlines (3 and 5 days), but only for the high procrastinator group. This is somewhat opposite to the common belief that people tend to procrastinate more on the tasks for which they have a longer time than those with the closest deadlines. On the contrary, low procrastinators evenly distributed their effort each day in the 3-day condition (without the intermediate peak in the 5-day condition). The time spent on the task and the “production” (keystrokes) showed identical patterns for both groups. This result does support our second hypothesis: shortening the deadline affects procrastinators’ and non-procrastinators productivity patterns differently. The peculiar pattern shown by high procrastinators is consistent, despite the time allowed to execute the task. In contrast, the low procrastinator seemed to adapt to the shortening of the deadline by evenly and diligently distributing their effort.

Previous research has suggested that procrastination is strictly related to temporal delay [61]. Thus, closer deadlines would be more effective in reducing procrastination because the temporal proximity of the rewards associated with task completion would help sustain task-related behavior. However, the behavioral pattern observed here remained consistent with both deadlines, suggesting that using a shorter deadline is not an effective way to deal with the tendency to engage in procrastination (albeit it leads to reaching the goal in a shorter time). Our interpretation of this result is that people who self-reported procrastination more may be characterized by a learning history of putting things off until the deadline is very close. On the contrary, people who self-reported procrastinating less may be characterized by a learning history of dealing with the task diligently and distributing their efforts across the available time. Both strategies may lead to reinforcement (a procrastinator is someone who postpones the execution of tasks, not someone who does not accomplish them) and may be equally distributed in the population. Indeed, our inspection of the individual productivity pattern shows that 28 subjects postponed writing to the last days, whereas the remaining 27 subjects worked from the beginning, reserving the last day(s) as a buffer period. This was half and half.

Another relevant finding concerns the role of task aversiveness in procrastination. Although previous research suggests that both procrastinators and non-procrastinators may perceive their tasks similarly [62], many studies suggest that procrastination could be used as a strategy to avoid negative feelings associated with the execution of a task [56]. In addition, early works on procrastination introduced the idea that people procrastinate on assignments due to their fear of failure and the motivation to protect their self-esteem [21]; see also [56]. Instead, in the present study, we find that the procrastinator is characterized by stable behavioral patterns unrelated to their intrinsic aversion to a task. This finding is compatible with what has been labeled “pacing style” [63,64], but we dare not make any assumptions regarding the existence of a specific mechanism (and its failure). Instead, considering that we perceive this phenomenon as a learning strategy used to manage deadlines, we examined whether the working patterns are the outcome of a more general type of reaction to stressful situations. For this reason, we measured the coping strategies commonly used to manage stressful events. Indeed, our third hypothesis was that different coping strategies would characterize procrastinators and non-procrastinators. Our results did not support this hypothesis as the two subgroups did not show any interaction regarding their coping styles. They were homogenous in terms of emotion-orientation and avoidance-orientation; they differed only in terms of task-orientation. Remarkably, the results show that low procrastinators report a more pronounced task-oriented coping, which is lacking in high procrastinators. This result shifts the focus of the discussion from what is wrong with procrastinators to what the protective factor that exists in non-procrastinators is. Apparently, the answer is that a pervasive coping strategy that is characterized by a focus on the task and efforts to solve stressful situations is incompatible with procrastination. Since no other differences were found between the two subgroups regarding the coping strategies, it seems that there is nothing special about procrastinators except the lack of such a task orientation.

In terms of work engagement and task performance, highly engaged individuals may be more likely to engage in task-oriented coping strategies and complete tasks efficiently and effectively. Organizational psychology research has consistently reported that work engagement is linked to desirable outcomes, such as job satisfaction, organizational commitment, and task performance [65,66]. Engaged employees are more invested in their work and feel a greater sense of responsibility toward their tasks and goals [66]. This heightened sense of ownership and accountability can inspire them to adopt task-oriented coping strategies, which involve proactively seeking solutions to problems and taking proactive steps to manage stress and workload [67,68]. However, the direction of these relationships is unclear: does work engagement result in adopting a task-oriented coping strategy, or (more plausibly) do coping strategies rooted in the individual’s learning history underlie a set of behaviors that we collectively call work engagement?

The present study has several strengths and some limitations. We examined procrastination by assessing a real-life task over time. Many studies on procrastination have focused their investigations exclusively on self-report measures or have correlated them with subjective responses to scenarios rather than actual behavior (see [64] for an extensive discussion). We decided to observe procrastination during the completion of an authentic task that is frequently assigned in academic and work settings familiar to students. Our analysis focused on continuous behavior rather than on a single-shot task where the time of execution is very close to its completion (e.g., sending a signed document, as in [53]). We thus wanted to focus on how people manage their effort and productivity when dealing with tasks and, therefore, investigate how they adjust their behavior according to settled deadlines. We relied on a self-report questionnaire to define two profiles (namely, high and low procrastinators). At the same time, we provided further evidence on the relation between PPS and actual procrastination behavior, thus corroborating the scale’s predictive validity.

Notwithstanding, in this study, we compared the performance of two subgroups of individuals categorized as high and low procrastinators based on their scores on a self-report measure. The problem with self-reports is that they may be biased by social desirability, particularly in evaluative contexts. Although questionnaires are a viable solution for investigating the differences in productivity, they are also quite different from comparing “real” procrastinators and non-procrastinators. A different strategy could be to assess the changes in performance that indicate a progressive productivity increase as the deadline approaches, and then categorize those subjects as procrastinators. Differences in self-reported procrastination might then be evaluated for the procrastinators and for the others. Although the differences between our two groups were statistically significant, 17 subjects of our pool whose PPS score was lower than the average value showed instead the typical procrastinator pattern (increased activity when close to the deadline). Data variability is endemic in psychological research, which is the point of using inferential statistics to assess differences between groups. Nevertheless, the fact that at the individual level, self-reports are somewhat separated from performance should make us think about the opportunity to focus on the conduct rather than the subjective accounts. 

A characteristic of this study was that our subjects were not allowed to miss the deadline: they needed to accomplish the task as a mandatory part of the course activity. This is the case in many work settings, but there are also other types of activity that are less stringent and for which missing the deadline and postponing the completion of a task is an option. Future studies should address the difference in the productivity time course between situations in which it is possible and not possible to miss the deadline. Many questions would be answered in such a setting. For example, in the present study, we have demonstrated that individuals with a PPS score above the average show a behavioral pattern of incremental productivity along the timeline; would the steepness of the linear relationship between productivity and the timeline decrease or increase in the case of a missed deadline? The experimental design employed here is easily implementable in academic and work settings. Assuming that the study/work materials are consistent with the type of study/work the participants are typically required to do, this design can be usefully implemented again for replication studies.

Other than those on the time course of the procrastination behavior, an essential finding of this study is that people show a consistent pattern independent of how distant the deadline is. This may have a practical implication, forcing procrastinators to complete tasks faster if the deadline is closer, even if they delay the onset. Indeed, using temporal buffers for managing delays is a common practice in Project Management (see [69]), and individual work is no exception. As a matter of fact, if your collaborators are procrastinators, it would not be a bad idea to tell them they have two days less to accomplish the task.

## Data Availability

The data presented in this study are available on request from the corresponding author.

## References

[1] Fee R.L., Tangney J.P. (2000). Procrastination: A means of avoiding shame or guilt?. J. Soc. Behav. Personal..

[2] Ferrari J.R., Pychyl T.A. (2000). Procrastination: Current Issues and New Directions.

[3] Ferrari J.R., Johnson J.L., McCown W.G. (1995). Procrastination and Task Avoidance: Theory, Research, and Treatment.

[4] Steel P. (2007). The nature of procrastination: A meta-analytic and theoretical review of quintessential self-regulatory failure. Psychol. Bull..

[5] Ferrari J.R. (2001). Procrastination as self-regulation failure of performance: Effects of cognitive load, self-awareness, and time limits on ‘working best under pressure’. Eur. J. Personal..

[6] Schraw G., Wadkins T., Olafson L. (2007). Doing the things we do: A grounded theory of academic procrastination. J. Educ. Psychol..

[7] Van Eerde W. (2003). A meta-analytically derived nomological network of procrastination. Personal. Individ. Differ..

[8] Sirois F.M., Melia-Gordon M.L., Pychyl T.A. (2003). “I’ll look after my health, later”: An investigation of procrastination and health. Personal. Individ. Differ..

[9] Balkis M., Duru E. (2016). Procrastination, self-regulation failure, academic life satisfaction, and affective well-being: Underregulation or misregulation form. Eur. J. Psychol. Educ..

[10] Tice D.M., Baumeister R.F. (1997). Longitudinal study of procrastination, performance, stress, and health: The costs and benefits of dawdling. Psychol. Sci..

[11] Stead R., Shanahan M.J., Neufeld R.W. (2010). “I’ll go to therapy, eventually”: Procrastination, stress and mental health. Personal. Individ. Differ..

[12] Maria-Ioanna A., Patra V. (2022). The role of psychological distress as a potential route through which procrastination may confer risk for reduced life satisfaction. Curr. Psychol..

[13] Klingsieck K.B., Grund A., Schmid S., Fries S. (2013). Why students procrastinate: A qualitative approach. J. Coll. Stud. Dev..

[14] Day V., Mensink D., O’Sullivan M. (2000). Patterns of academic procrastination. J. Coll. Read. Learn..

[15] Unda-López A., Osejo-Taco G., Vinueza-Cabezas A., Paz C., Hidalgo-Andrade P. (2022). Procrastination during the COVID-19 pandemic: A scoping review. Behav. Sci..

[16] Hoover E. (2005). Tomorrow, I Love Ya!. Chron. High. Educ..

[17] Barel E., Shahrabani S., Mahagna L., Massalha R., Colodner R., Tzischinsky O. (2023). State Anxiety and Procrastination: The Moderating Role of Neuroendocrine Factors. Behav. Sci..

[18] Zarrin S.A., Gracia E., Paixão M.P. (2020). Prediction of academic procrastination by fear of failure and self-regulation. Educ. Sci. Theory Pract..

[19] Hall N.C., Lee S.Y., Rahimi S. (2019). Self-efficacy, procrastination, and burnout in post-secondary faculty: An international longitudinal analysis. PLoS ONE.

[20] Suárez-Perdomo A., Ruiz-Alfonso Z., Garcés-Delgado Y. (2021). Profiles of undergraduates’ networks addiction: Difference in academic procrastination and performance. Comput. Educ..

[21] Solomon L.J., Rothblum E.D. (1984). Academic procrastination: Frequency and cognitive-behavioral correlates. J. Couns. Psychol..

[22] Ferrari J.R., Emmons R.A. (1995). Methods of procrastination and their relation to self-control and self-reinforcement: An exploratory study. J. Soc. Behav. Personal..

[23] Ferrari J.R. (1991). Procrastination and project creation: Choosing easy, nondiagnostic items to avoid self-relevant information. J. Soc. Behav. Personal..

[24] Ferrari J.R. (1992). Procrastination in the workplace: Attributions for failure among individuals with similar behavioral tendencies. Personal. Individ. Differ..

[25] Ferrari J.R. (1994). Dysfunctional procrastination and its relationship with self-esteem, interpersonal dependency, and self-defeating behaviors. Personal. Individ. Differ..

[26] Milgram N.N., Mey-Tal G., Levison Y. (1998). Procrastination, generalized or specific, in college students and their parents. Personal. Individ. Differ..

[27] Lay C.H. (1997). Explaining lower-order traits through higher-order factors: The case of trait procrastination, conscientiousness, and the specificity dilemma. Eur. J. Personal..

[28] Dewitte S., Schouwenburg H.C. (2002). Procrastination, temptations, and incentives: The struggle between the present and the future in procrastinators and the punctual. Eur. J. Personal..

[29] Gustavson D.E., Miyake A., Hewitt J.K., Friedman N.P. (2014). Genetic relations among procrastination, impulsivity, and goal-management ability: Implications for the evolutionary origin of procrastination. Psychol. Sci..

[30] Harriott J.S., Ferrari J.R., Dovidio J.F. (1996). Distractibility, daydreaming, and self-critical cognitions as determinants of indecision. J. Soc. Behav. Personal..

[31] Rebetez M.M.L., Rochat L., Van der Linden M. (2015). Cognitive, emotional, and motivational factors related to procrastination: A cluster analytic approach. Personal. Individ. Differ..

[32] Bandura A. (1997). Self-Efficacy: The Exercise of Control.

[33] Judge T., Bono J. (2001). Relationship of core self-evaluations traits-self-esteem, generalized self-efficacy, locus of control, and emotional stability-with job satisfaction and job performance: A meta-analysis. J. Appl. Psychol..

[34] Janssen T., Carton J.S. (1999). The Effects of Locus of Control and Task Difficulty on Procrastination. J. Genet. Psychol..

[35] Milgram N., Marshevsky S., Sadeh C. (1995). Correlates of academic procrastination: Discomfort, task aversiveness, and task capability. J. Psychol..

[36] Ackerman D.S., Gross B.L. (2005). My instructor made me do it: Task characteristics of procrastination. J. Mark. Educ..

[37] Schaufeli W.B., Salanova M., González-Romá V., Bakker A.B. (2002). The measurement of engagement and burnout: A two sample confirmatory factor analytic approach. J. Happiness Stud..

[38] Bakker A.B., Demerouti E., Schaufeli W.B. (2012). The crossover of work engagement between working couples: A closer look at the role of empathy. J. Manag. Psychol..

[39] Kim H.J., Shin K.H., Kang S.W. (2021). An investigation of the relationship between work engagement and job satisfaction of fashion industry employees. J. Fash. Mark. Manag..

[40] Saks A.M., Gruman J.A. (2014). What do we really know about employee engagement?. Hum. Resour. Dev. Q..

[41] Chun Chu A.H., Choi J.N. (2005). Rethinking procrastination: Positive effects of “active” procrastination behavior on attitudes and performance. J. Soc. Psychol..

[42] Choi J.N., Moran S.V. (2009). Why not procrastinate? Development and validation of a new active procrastination scale. J. Soc. Psychol..

[43] Hensley L.C. (2014). Reconsidering active procrastination: Relations to motivation and achievement in college anatomy. Learn. Individ. Differ..

[44] Schouwenburg H.C., Lay C.H. (1995). Trait procrastination and the big-five factors of personality. Personal. Individ. Differ..

[45] Schouwenburg H.C., Groenewoud J. (2001). Study motivation under social temptation; effects of trait procrastination. Personal. Individ. Differ..

[46] Ainslie G. (1975). Specious reward: A behavioral theory of impulsiveness and impulse control. Psychol. Bull..

[47] Kirby K.N. (1997). Bidding on the future: Evidence against normative discounting of delayed rewards. J. Exp. Psychol. Gen..

[48] Laibson D. (1997). Golden eggs and hyperbolic discounting. Q. J. Econ..

[49] Rothblum E.D., Solomon L.J., Murakami J. (1986). Affective, cognitive, and behavioral differences between high and low procrastinators. J. Couns. Psychol..

[50] Moon S.M., Illingworth A.J. (2005). Exploring the dynamic nature of procrastination: A latent growth curve analysis of academic procrastination. Personal. Individ. Differ..

[51] Zhang S., Feng T. (2020). Modeling procrastination: Asymmetric decisions to act between the present and the future. J. Exp. Psychol. Gen..

[52] Wessel J., Bradley G.L., Hood M. (2019). Comparing effects of active and passive procrastination: A field study of behavioral delay. Personal. Individ. Differ..

[53] Zuber S., Cauvin S., Haas M., Daviet A.S., Da Silva Coelho C., Kliegel M. (2020). Do self-reports of procrastination predict actual behavior?. Int. J. Methods Psychiatr. Res..

[54] Svartdal F., Steel P. (2017). Irrational delay revisited: Examining five procrastination scales in a global sample. Front. Psychol..

[55] Endler N.S., Parker J.D.A. (1990). Coping Inventory for Stressful Situations (CISS): Manual.

[56] Steel P. (2010). Arousal, avoidant and decisional procrastinators: Do they exist?. Personal. Individ. Differ..

[57] Svartdal F., Pfuhl G., Nordby K., Foschi G., Klingsieck K.B., Rozental A., Carlbling R., Lindblom-Ylänne S., Rębkowska K. (2016). On the measurement of procrastination: Comparing two scales in six European countries. Front. Psychol..

[58] Sirigatti S., Stefanile C. (2009). CISS—Coping Inventory for Stressful Situation Stanrdizzazione e Validazione Italiana.

[59] Reichheld F.F. (2003). The one number you need to grow. Harv. Bus. Rev..

[60] Reichheld F.F., Covey S.R. (2006). The Ultimate Question: Driving Good Profits and True Growth.

[61] Sirois F., Pychyl T. (2013). Procrastination and the priority of short-term mood regulation: Consequences for future self. Soc. Personal. Psychol. Compass.

[62] Ferrari J.R., Mason C.P., Hammer C. (2006). Procrastination as a predictor of task perceptions: Examining delayed and non-delayed tasks across varied deadlines. Individ. Differ. Res..

[63] Blount S., Janicik G.A., Neale M.A., Mannix E., Sondak H. (2002). Getting and staying in-pace: The in-synch preference and its implications for work groups. Research on Managing Groups and Teams.

[64] Gevers J., Mohammed S., Baytalskaya N. (2015). The zconceptualization and measurement of pacing styles. Appl. Psychol..

[65] Bakker A.B., Demerouti E. (2017). Job demands–resources theory: Taking stock and looking forward. J. Occup. Health Psychol..

[66] Harter J.K., Schmidt F.L., Hayes T.L. (2002). Business-unit-level relationship between employee satisfaction, employee engagement, and business outcomes: A meta-analysis. J. Appl. Psychol..

[67] Searle B.J., Auton J.C. (2015). The role of perceived workplace support, distributive justice and motivation in workplace absenteeism. J. Bus. Psychol..

[68] Gargantini T., Daly M., Sherlock J., Lazebnik T. (2022). Providing Safe Space for Honest Mistakes in the Public Sector Is the Most Important Predictor for Work Engagement after Strategic Clarity. Sustainability.

[69] Project Management Institute (2022). A Guide to the Project Management Body of Knowledge (PMBOK Guide).

